# User Experiences of the McMaster Optimal Aging Portal’s Evidence Summaries and Blog Posts: Usability Study

**DOI:** 10.2196/humanfactors.6208

**Published:** 2016-08-19

**Authors:** Angela M Barbara, Maureen Dobbins, R Brian Haynes, Alfonso Iorio, John N Lavis, Anthony J Levinson

**Affiliations:** ^1^ Health Information Research Unit Department of Clinical Epidemiology and Biostatistics McMaster University Hamilton, ON Canada; ^2^ School of Nursing McMaster University Hamilton, ON Canada; ^3^ McMaster Health Forum, Centre for Health Economics and Policy Analysis Department of Clinical Epidemiology and Biostatistics McMaster University Hamilton, ON Canada; ^4^ Division of e-Learning Innovation Faculty of Health Sciences McMaster University Hamilton, ON Canada

**Keywords:** Web-based health information, consumer health information, usability testing, knowledge translation, aging

## Abstract

**Background:**

Evidence summaries and blogs can support evidence-informed healthy aging, by presenting high-quality health research evidence in plain language for a nonprofessional (citizen) audience.

**Objective:**

Our objective was to explore citizens’ perceptions about the usability of evidence summaries and blog posts on the Web-based McMaster Optimal Aging Portal.

**Methods:**

Twenty-two citizens (aged 50 years and older) and informal caregivers participated in a qualitative study using a think-aloud method and semistructured interviews. Eleven interviews were conducted in person, 7 over the telephone, and 4 by Skype.

**Results:**

We identified themes that fell under 4 user-experience categories: (1) desirability: personal relevance, (2) understandability: language comprehension, grasping the message, dealing with uncertainty, (3) usability: volume of information, use of numbers, and (4) usefulness: intention to use, facility for sharing.

**Conclusions:**

Participants recognized that high-quality evidence on aging was valuable. Their intended use of the information was influenced by how much it applied to their own health circumstances or those of a loved one. Some specific formatting features that were preferred included consistent layout, content organized by subheadings, catchy titles, numerical information summarized in a table, and inclusion of a glossary.

## Introduction

### Background

At a time when patients have become more active participants in health care decision making [1], the Internet can be used as a healthy-aging information “tool” [2,3]. Increasingly, people turn to the Internet as a source of information, motivation, and support for healthy living and management of common health conditions [4]. Accessing Web-based health information helps older people to take better care of their own and loved ones’ health, either by attending to an existing health condition or improving health behaviors [5]. Seniors can also use the information to prepare for and follow-up after a health care–related appointment [5]. For patients who want to ask questions that they perceive as embarrassing or private, the Internet provides anonymity and convenience [6]. Many older adults are also supported by family and informal caregivers who seek out Web-based health information on their behalf [2,7]. However, much of the health information available on the Internet has not been informed by good-quality evidence [8,9], and therefore is unlikely to produce beneficial results on health.

### McMaster Optimal Aging Portal

A full description of the Web-based McMaster Optimal Aging Portal [10] and its components is available elsewhere [11]. In this paper, we focus on 2 types of Portal content that provide citizen-friendly research evidence about aging: “lay” evidence summaries and blog posts about the best available research. ‘Citizens’ include members of the general public and health care consumers such as patients and caregivers. The term is used to distinguish them from health care professionals (clinicians, public health workers, policymakers) who are the typical target audiences for research evidence.

A scoping review found a scarcity of knowledge translation research focused on the care of older adults [12]. Evidence summaries and blogs can support evidence-informed healthy aging. Within the knowledge-to-action cycle framework, these resources fall into the third milestone of adapting knowledge to the local context [13], by explaining and translating health research evidence into plain or lay language for citizens. While we know older adults and caregivers are going to the Internet to find health information [14-16], we need to know about the optimal ways to package that information to be most useful [17].

As part of the overall formative evaluation of the Portal [11], we conducted individual interviews with citizens to identify prominent perceptions about the usability of the evidence summaries and blog posts.

## Methods

### Evidence Summaries and Blog Posts

**Figure 1:**
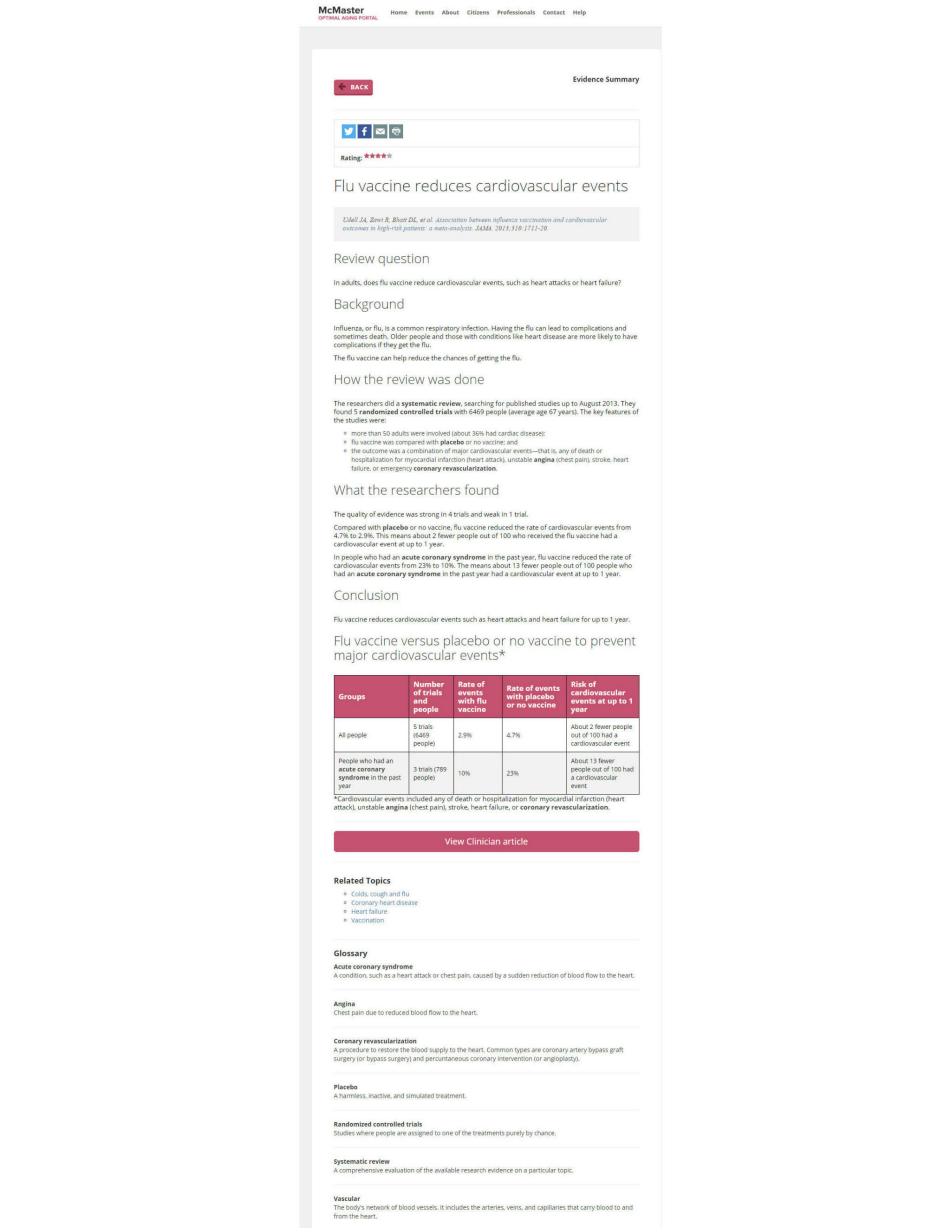
Evidence summary on the McMaster Optimal Aging Portal.

A full account of the evidence summaries and blog posts are published elsewhere [11]. In short, evidence summaries describe the findings from the best available research (typically, systematic reviews) on a particular topic in plain language. The research comes from 3 professional databases containing systematic reviews and individual studies that have been critically appraised for scientific merit: McMaster Premium LiteratUre Service (McMasterPLUS) [18,19], Health Evidence [20], and Health Systems Evidence [21,22]. To be included in the Portal, the content must be relevant to healthy aging and health care for older people. The summaries are written by trained Portal research staff, who each have graduate degrees in health research methodology or a related field. They are organized into the following sections: declarative title, descriptive title, subject of the study, research question, background, how the review (research) was done, what the findings are, and definitions of key technical terms (Figure 1).

**Figure 2:**
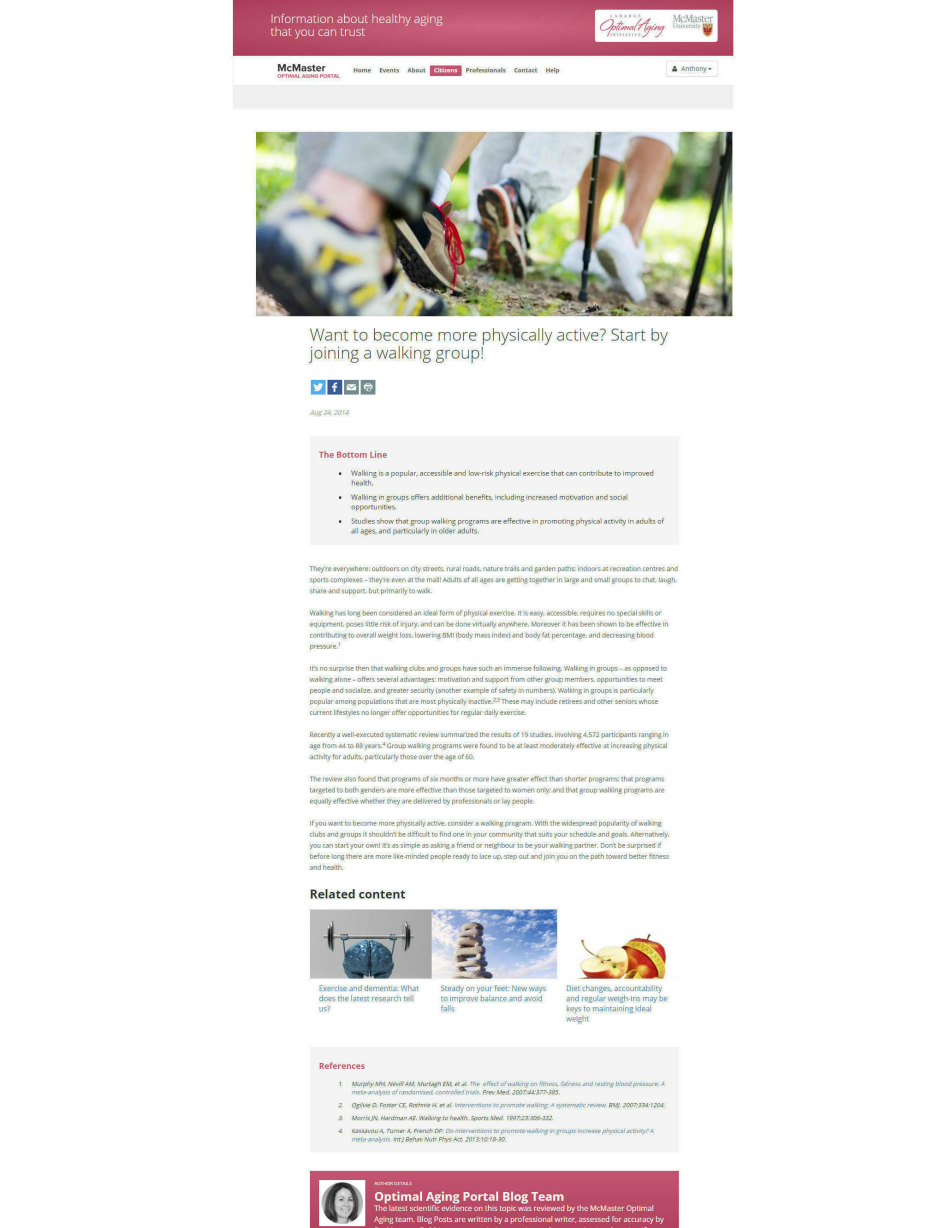
Blog post on the McMaster Optimal Aging Portal.

Blog posts are discussions or commentaries on the best available, recent scientific evidence specific to healthy aging. The topics were determined by consensus of the Portal’s expert advisory committee. The committee consists of professionals with expertise in diverse fields, such as aging, epidemiology, geriatrics, health policy, health informatics, and rehabilitation. Blog posts typically contain the following: feature image about the topic being discussed, text about the topic’s importance, research on the topic, why the research findings are important, bottom line messages, references, links to other relevant blogs or items on the Portal, and author details. The writer of the blog post is chosen on a case by case basis. Blogs that cover a specific topic area (eg, sleep disorders, cognitive functioning) are written by an invited scientist or practitioner that is an expert in that field. Some blogs focus on the research featured in an evidence summary; these are written by a professional writer and reviewed for accuracy by a content expert. Both types of blogs are edited by a professional editor (Figure 2).

### Participants

We used purposive sampling to form a sample composed of (1) citizens aged 50 years and older, and (2) informal caregivers (persons who provide unpaid care to an older parent/family member/friend/loved one). Participants were required to have access to a computer with an Internet connection.

Recruitment was done in conjunction with usability testing of the entire website [11]. We distributed and posted advertisements for both projects through local community and academic networks (4 academic or research group listservs, 3 recreation centers for seniors, 1 retired community, 1 professional organization, and informal contacts through members of the Portal team). Interested individuals contacted the interviewer. After screening for eligibility, participants were emailed confirmation of the interview details and the consent form.

### Procedures

One author (AMB) conducted all the one-on-one interviews, either in person (in a laboratory on the McMaster University campus), by telephone, or using Skype, based on participant preference. The choice to review summaries or blogs was also made by participants. The concepts of evidence summaries or blog posts were introduced to participants using the copy available on the Portal. Participants were instructed to choose what to review from a list of selected evidence summaries or blogs available on the Portal.

We used the think-aloud method [23], whereby users verbalize their thoughts as they read through the summaries or blogs. Participants were probed if they became quiet (eg, “What are you thinking?” “What are you looking at?” “What do you think about what you are reading?”) Then, a semistructured interview guide (Multimedia Appendix 1) was used to elicit further feedback, based on a modified version of Morville’s User Experience Honeycomb [24,25], whereby the following elements of information create a valuable user experience: findable, accessible, desirable, understandable, usable, credible, and useful. Interview questions included: “Why did you choose this one to review?” “Have you been looking for anything like this?” “What do you think of how the information was presented?” “How clear was the information?” “If you found this on your own, what would you do with it?” Following the interviews, participants were asked sociodemographic questions.

### Data Analysis

Interviews were audiotaped, transcribed, and checked for accuracy. We used a framework approach, encompassing both thematic analysis and case analysis [26,27]. Thematic categories and patterns were compared between and within participants and linked from the identified theme to the original data. A framework approach was used because we had clear research goals in advance, but also wished to identify new themes emerging from the data. A subset of interviews were dual-coded by 2 authors (AMB, AJL), who met regularly to discuss coding, indexing, and interpretation of the results. We organized themes according to Morville’s user-experience elements. QSR NVivo 9 software was used for coding and data management.

Ethical approval was granted by the Hamilton Integrated Research Ethics Board. This work was supported through the Labarge Optimal Aging Initiative.

## Results

### Study Participants

Sixty-three people contacted the interviewer in response to study advertisements. Fifteen respondents (15/63, 24%) participated in usability of the overall Portal, but did not evaluate the evidence summaries or blogs. Twenty-six (26/63, 41%) people were excluded from participation: 17 were considered noncitizens (ie, clinicians or public health professionals), 7 had scheduling conflicts, and 2 respondents did not use computers.

Twenty-two participants (22/63, 35%) were included in the following study. Eleven people (11/22, 50%) chose to evaluate evidence summaries, 7 (7/22, 32%) chose to evaluate blog posts, and 4 people (4/22, 18%) volunteered to review both. Twenty summaries and 14 blogs (11 written by experts and 3 written by the professional writer) were evaluated by at least 1 participant (Table 1).

The sample consisted of 12 citizens and 10 other citizens that were also informal caregivers (Table 2). Citizens were retired and all but 1 person reported having a health condition. Caregivers were mostly women (all but 1) and younger in age compared with noncaregivers (mean years, 58 vs 75). Each participant was given a study identification, which follows their quotes in the findings.

All participants were recruited from the Hamilton area in Ontario, Canada between July and September 2014. Sessions lasted from 30 to 67 minutes (mean = 43). Eleven interviews were conducted in person, 7 over the telephone, and 4 by Skype.

**Table 1:** Sample of evidence summaries and blog posts reviewed.

Content type	Health area	Title
**Evidence summaries**		
	Exercise	Yoga reduces pain and disability at up to 1 year in people with low back pain
	Memory and cognition	Tests detect dementia in older people; cognitive stimulation or some drugs may slightly improve cognitive function
	Heart disease	Multiple lifestyle changes in people with established coronary heart disease reduce the risk for cardiovascular events
	Health information technology	Computer-delivered interventions have a small effect on knowledge and some health behaviors
	Testing and treatment decisions	Unnecessary medication use in frail older adults can be reduced through team-based care, providing education to providers and reviewing prescribing practices
	Psychological and mental health	Meaningful social roles may improve health and well-being for people in retirement
**Blog posts**		
	Exercise	How fast should I walk to cross the road safely? Fast facts about walking speed
	Nutrition	Does salt really affect blood pressure?
	Memory and cognition	Treating behavioral problems of dementia: when confusion leads to controversy
	Social health	Loneliness hurts. How to recognize loneliness as a health concern
	Sleep disorders	Sleep and aging: how many zzz's are optimal to stay healthy?

**Table 2:** Participant demographic characteristics.

Group	Study ID	Age	Gender	Employment status	Health status	Education
**Citizen**						
	I-01	62	Female	Retired	Healthy	Some post-graduate
	I-02	66	Female	Retired	One or more health conditions	Some college/university
	I-03	66	Female	Retired	One or more health conditions	Some college/university
	I-04	70	Male	Retired	One or more health conditions	Post-graduate
	I-05	74	Male	Retired	One or more health conditions	Some college/university
	I-06	76	Female	Retired	Healthy	College/university
	I-07	79	Male	Retired	One or more health conditions	Post-graduate
	I-08	80	Female	Retired	One or more health conditions	College/university
	I-09	81	Female	Retired	One or more health conditions	College/university
	I-10	82	Male	Retired	One or more health conditions	Post-graduate
	I-11	84	Male	Retired	One or more health conditions	Some college/university
	I-12	84	Male	Retired	One or more health conditions	Post-graduate
**Caregiver**						
	A-13	23	Female	Part-time work, part-time student	Healthy	College/university
	A-14	48	Female	Full-time work	Healthy	Post-graduate
	A-15	55	Female	Retired	Healthy	College/university
	A-16	59	Female	Full-time work	Healthy	College/university
	A-17	60	Female	Part-time work	Healthy	High school
	A-18	60	Male	Retired	One or more health conditions	Post-graduate
	A-19	67	Female	Retired	One or more health conditions	College/university
	A-20	67	Female	Retired	Healthy	College/university
	A-21	70	Female	Retired	Healthy	Post-graduate
	A-22	75	Female	Retired	Healthy	College/university

**Table 3:** Findings, organized into 4 aspects of the user experience, themes, and subthemes.

User experience element and explanation	Theme
Desirability: users feel the product is worth having and have a positive emotional response to it	Personal relevance
Understandability: users comprehend both what kind of product it is and its content	Language comprehension; grasping the message; dealing with uncertainty
Usability: users can use the product easily, effectively, and with satisfaction	Volume of information; use of numbers
Usefulness: users find the product has practical value	Intention to use the information; facility for sharing

### Findings

For this study, we describe the themes that fall under 4 user-experience categories (Table 3). Findability, accessability, and credibility are also important facets of the user experience, but have been discussed elsewhere as part of the usability of the overall Portal [11]. For additional exemplar quotes, please see Multimedia Appendix 2.

#### Desirability

##### Personal Relevance

Universally, participants selected a resource to review because the title contained a topic that was personally significant or applicable. Participants were concerned about a condition or situation that they were presently dealing with, had previously dealt with, or anticipated they would face in the future. Eight citizens and 8 caregivers were specifically drawn to topics that affected loved ones.

I often look online for stuff about this for over the last about 11 years or so, since 2003, about diabetes and exercise and I have been looking online recently for what I key in is osteoarthritis. My mother had osteoarthritis towards the end of her life. I have developed a little bit, not bad yet, so I look up ways to deal with osteoarthritis and diabetes.

A-18

During the interview, 7 people specifically acknowledged the importance of aging on health and, overall, people responded positively to the Portal resources. In general, readers wanted information related to a specific topic and were less concerned about the type or format of the content (evidence summary, blog post, or other). However, 7 people wanted to understand what the summaries and blogs were supposed to be so they could read the information in the appropriate context.

Seven participants wanted to read a summary or blog because its topic was perceived as an important social issue or was featured recently in the media.

I was listening to a program on the radio about social isolation. And it found that when people are in a neighborhood where they feel safe and are familiar with, the general health of the elderly was much better, even in terms of lower heart attacks and stuff like that.

A-20

Engagement or absorption with the material was often demonstrated when 13 participants paused during reading to tell a personal anecdote or story. Two people claimed they would only read segments that were personally relevant and skim or skip the rest. Some participants related the information to their own situation by paraphrasing what they read. Sixteen users reacted emotionally (eg, reassured, alarmed, surprised) to what they read, especially by study conclusions.

Wonderful, the results are good news!

I-01

Oh shit! Really? So that would scare me because I have a problem keeping my sleep patterns normalized.

A-16

Users’ prior knowledge about a subject also influenced the desire to read the information. Those who knew little were interested to find out more by reading carefully compared with participants who felt they were already well-versed about the topic and scanned the information. Four users chose a resource to learn more about an unfamiliar medical concept (eg, multimorbidity, psychotropics).

Each summary has a declarative title, stating the key result(s) of the study or systematic review succinctly. Seven people felt these titles were long and difficult to understand or “mouthfuls.” Having a title that “grabs a reader” was seen as important, whereas the declarative titles were “not enticing.” Some users felt the title was a “spoiler,” which did not motivate them to read the content.

The title sounds like the conclusion. I would rather have a title that was more descriptive as to what I could expect in the article. This one is a bit disappointing. It really does not tell you much more than what is in the title.

I-01

One user assumed the titles of the summaries were the original article titles. On the other hand, 2 people commented that the blog titles were appealing and “catchy.”

#### Understandability

##### Language Comprehension

Twelve participants, some of whom had some familiarity with research or the medical profession, thought the information in the evidence summaries was clear and easy to understand. In contrast, 8 participants felt that the summaries were written “by professionals for professionals” and questioned whether citizens would fully understand them.

It looks as if it is meant for professionals because of the wording. I think if you are aiming at older people, you don’t want it to be patronizing but I think slightly less scientific wording would be more attractive.

A-22

When the cursor hovers over a bolded term in the body of an evidence summary, a pop-up box with the definition appears. This feature was received positively, as was the inclusion of the glossary at the end of some summaries, especially as most people were uncertain of the meanings of words such as ‘systematic review’ and ‘randomized controlled trial’. Four participants recommended that a glossary be added to all summaries and also to blog posts. Some wanted the glossary to be expanded to include other scientific terminology, such as ‘intervention’, ‘outcome’, ‘control (group)’, ‘quality of evidence’, and ‘meta-analysis’. Some participants struggled with certain phrases (eg, “range within which the average value might fall”) and medical concepts (eg, dementia vs cognitive impairment). Many were unfamiliar with professional organizations (eg, Cochrane Collaboration), measurement instruments (eg., AMSTAR tool), and specific medications (eg, Nonsteroidal anti-inflammatory drugs or NSAIDs) or herbal products (eg, gingko biloba), unless they were taking them or they had been featured in the news (eg, Celebrex). In total, 17 participants identified terminology with which they were not completely familiar.

##### Grasping the Message

Eleven participants understood the key message(s) by looking at the conclusions in the evidence summaries or the "Bottom Line" in the blogs. At least 5 people said they would look at these sections first. Some wanted to re-read the resource more closely once they scanned it to comprehend the message. Participant A-15 stated “So a part of my habit is to always just to skip up and down and just to kind of get an overall view before I dig into an article.”

Five people read parts of the text (sentence or paragraph) a second time (aloud or to themselves) to make sure they understood what they were reading. Fourteen respondents looked to the facilitator for confirmation that they understood the summary’s meaning. “What the researchers found” was felt to contain the most important piece of information. “How the review was done” was of least interest. Four individuals wanted to know more about the actual intervention. According to participant A-17, “I thought it would give you the exercises or some examples of what not to do.”

Users were very satisfied if the intervention-related studies were described in enough detail that they might implement them (eg, specific drugs to discuss with their physician; small group activities to stimulate thinking and memory).

##### Dealing With Uncertainty

Two participants were cognizant that research did not always provide clear cut answers or provide “a magic pill.” However, another 2 were exasperated. After reading that the research findings were not certain, participants were unsure why the information was presented. One participant reacted “Now, I am cranky,” and explained further:

“Bottom line, the research shows that the amount of sleep, the quality of your sleep may change as we age.” That means nothing to me. That’s the kind of thing that a person tells you when they don’t want to tell you anything important. So I understand that that’s what the research says, but it’s frustrating because I read to the end of this blog and there are no answers to my questions. I am getting gobbledygook. It started with a question and ends with a question.

A-16

#### Usability

##### Volume of Information

Eleven people who evaluated the evidence summaries felt the one page had “just the right amount of information.” Some readers did not notice the length, rather they pointed out that the standardized format and layout made the content easy to read. The shortness of the summary guaranteed that most people would actually read it rather than only skimming it. Links to related content were appreciated by those who were interested in additional information.

They do not get into a ton of details, but I think that that is what some people are looking for. They are just looking for a kind of a summary and recap, or an introduction to some of these things.

A-13

Perceptions about the length of the blogs varied. Some commented that they were approximately the length of a magazine article, which was appropriate. However, at least 2 people felt they were too lengthy. Satisfaction with length was often associated with engagement; that is, 7 users did not mind reading longer articles or even remark on length if they were engrossed by the content.

##### Use of Numbers

Overall, 11 participants were happy with the presentation of data in table format (eg, summing up the findings of a systematic review) and found it informative. One participant felt,

“the table is easier to grasp than reading lines and lines of information”

A-17

Eleven people looked for demographic information (eg, ages of research participants); and 7 people looked for sample size (eg, number of participants in the systematic reviews or studies, number of studies included in the systematic reviews). Three participants said they liked the use of percentages.

I want to know how effective something is, quantification. Numbers help make things clearer and more useful.

A-18

On the other hand, 2 participants claimed they were not “number people” and preferred the focus to be on individuals (eg, how many people were helped rather than percentages or statistics). Four people wanted nonscientific information.

I am a person, not a statistic. So, I wanted to know the anecdotal evidence, because I could be the person outside the standard deviation. As a human being, all I care about is: Will this affect me? Will it hurt me?

A-16

#### Usefulness

##### Intention to Use the Information

Ten participants were satisfied with resources if they learned something new: “I didn’t know exercise could help me with dementia” [I-02]. Others were pleased to reconcile any new information with their existing knowledge or understanding.

As mentioned above, many felt a discrepancy between the type of information available and their information needs. Eight participants wanted more detailed practical information that could be applied to improve their own health (especially regarding treatment or preventative activities).

The one thing that I would want is what should I do differently? This one has nothing about that. They didn’t actually talk about the interventions. I would have been interested in knowing what they were. So I didn’t learn anything… a little disappointing.

I-01

The resource was useful if the information could be applied to their demographic or personal situation. If the information was indeed relevant, 10 participants intended to apply it. Participant I-09 felt that “the information is good in that it gives me some choices and the pros and cons; and then it is up to me.”

Some readers felt they would have benefitted if they had access to the information when they were dealing with a past situation (eg, making treatment decisions, dealing with the diagnosis in a parent). Others felt the summaries would be useful for future reference.

I chose this one about fall prevention because my grandmother fell and broke her hip, and my mother fell and broke her hip, I am assuming that is probably what will happen to me.

I-02

##### Facility for Sharing

Thirteen participants were keen to discuss the information with family and friends, and were pleased that the resource itself could be easily shared: “I know a lot of people with sleep apnea who don’t realize they have it, and would share this with them” [A-16].

Five participants wanted to discuss the applicability of the information with their health care provider. Several felt that Portal resources should be available through physician offices and other health care settings. One participant decided: “I will ask my doctor about whether there are any decision aids available for me” [A-21].

## Discussion

### Principle Findings

This study was conducted to better understand citizens’ perceptions of the desirability, understandability, usability, and usefulness of the evidence summaries and blog posts available on the McMaster Optimal Aging Portal. By studying user’s impressions, we can improve the translation of research evidence for citizens.

Participants recognized that high-quality evidence on aging was valuable. Their intended use of the information was influenced by how much it applied to their own health circumstances or those of a loved one. Participants wanted to read information about a specific health topic regardless if it was presented as a summary or blog. Nevertheless, specific formatting features were preferable (eg, consistent layout, content organized by subheadings, catchy titles of the blogs vs the declarative titles of the summaries). Participants wanted a narrative summary and information on how many people were helped or harmed by the intervention. Numerical information was preferably summarized in a table. While many participants were unfamiliar with research or medical terminology, there was a desire to learn it as demonstrated by the enthusiastic response to the glossary.

The study also suggested several challenges in presenting research evidence to citizens. Several participants perceived the evidence summaries to be written for professionals rather than a citizen audience. This suggests that, despite deliberate efforts of the Portal team to simplify the language, the information remained complex in the eyes of some. Systematic reviews typically investigate the effectiveness of an intervention in a specified population (eg, how effective are interventions with multiple lifestyle components in reducing the risk for cardiovascular events in patients with established heart disease?) However, patients and caregivers want to know how statistical results should be translated for individuals.

Participants often absorbed the evidence in the context of their own or other peoples’ experiences. Some users were puzzled or frustrated by research with weak evidence or that did not have definitive conclusions. This highlights the need for instructional resources for citizens to learn that uncertainty is always present in health research (eg, a primer, meaningful graphics, or other multimedia formats to facilitate learning about research methods). Others have recommended the use of personal narratives to elucidate research outcomes [28].

### Comparison With Previous Work

The findings of this study are in accordance with previous studies on the presentation of health information. The Cochrane Collaboration tested their Plain Language Summaries with citizens [29,30]. They also found a lack of familiarity with research-based concepts and individual variation in how users wanted research findings to be displayed (ie, text or numbers, or both). Like our study, their participants also wanted quantitative results to be presented in a table. They also preferred summaries divided by headings; and preferred headings in question format, which is similar to our participants’ suggestions that the titles of the Portal summaries be in the form of an appealing question.

Work in disease-specific settings has found that seekers of Web-based health information have similar needs as our study participants. For example, people with multiple sclerosis also desire information that is personally applicable and educational tools (such as a glossary and methodological information), had emotional responses to information, and wanted integrated Web-based information with existing knowledge or information from other channels [31,32]

One page was perceived as an ideal length for the Portal evidence summaries. This is reinforced by consistent study findings that too much information can reduce comprehension [33]. Similar to our findings about blog length, other research has found that citizens differ on their notions of how much information is too much based on their preferences and needs [33].

The “fuzzy trace theory” of medical decision making argues that people want the gist of information and its bottom-line meaning as opposed to the literal details [34]. Our study observations support this theory in that our participants appreciated that information was presented in “chunks,” which reduced cognitive load and allowed them to concentrate on specific chunks (ie, what the researchers found) and scan the remaining content.

Our findings also agree with survey research indicating that approximately one-third of older adults will talk about the health information they obtained from the Internet with their doctor [35]. Studies have found that patients will prepare for a doctor’s visit by looking for health information [5]. Information access allows patients to evolve from passive recipients to active partners in their health care, and clinicians to transform from having an authoritarian role to being a partner in the care of their patients [16]. Physician encouragement and guidance regarding Internet usage by patients can also improve patient-physician communication [36]. Therefore, having high-quality evidence summaries and blog posts can empower patients, resulting in better discussions during clinical consultations and higher patient satisfaction.

Other studies have also found that citizens have difficulty in applying the findings of systematic reviews or individual research studies to their own individual situation [28,37,38]. While many users felt it was useful to be informed of current research in aging, at least one-third were looking for information that would help them make a personal decision, especially regarding treatment. We are currently exploring the addition of resources that will assist citizens in implementing research findings while addressing their values and preferences; specifically, patient decision aids and patient versions of clinical practice guidelines.

### Limitations

We did not test a random sample, which may affect the generalizability of the findings. Participants were well-educated. Our testing occurred in an artificial setting; participants were not accessing or reading the Portal resources in the context that is expected, and this could have affected responses. We did not formally assess health literacy, which is especially pertinent for older adults and will have affected how individuals processed and understood the information [39,40].

### Conclusions

We identified factors that influence the usability of the Portal evidence summaries and blog posts. These factors will be used to improve the content and design templates for development of future summaries and blogs. To feature the Bottom Line more prominently, it will moved from the end of each blog post to the beginning. Because participants made a decision about whether to print or share a blog once they had read it, we will add the “sharing” buttons (now featured only at the top of the blog page) at the end of the blog as well. A prompt to describe an evidence summary for novice users will be added to the top of the Web page. At the end of an evidence summary, we will include additional related content on the Portal, such as “Related Evidence Summaries” and “Related Web Resources.” Future research will focus on the impact of the enhanced formats on understanding, applicability, decision-making, and behavior of both citizens and health professionals in real-life settings.

## Appendix

Multimedia Appendix 1 Interview guide for evaluation of evidence summaries / blog posts.

Multimedia Appendix 2 Themes and quotes related to elements of the user experience.
