# Supplementary material for: User Experiences of the McMaster Optimal Aging Portal’s Evidence Summaries and Blog Posts: Usability Study
Source: JMIR Hum Factors. 2016 Aug 19;3(2):e22. doi: 10.2196/humanfactors.6208 (PMC5010647; doi:10.2196/humanfactors.6208)
Supplement: Multimedia Appendix 1 [file humanfactors_v3i2e22_app1.pdf]

User Experiences of the McMaster Optimal Aging Portal's Evidence Summaries and Blog Posts: Usability Study

Appendix 1:

Interview Guide for Evaluation of Evidence Summaries / Blog Posts

Introduction

Thank you for participating in this study. Your feedback will help us improve the information presented on the McMaster Optimal Aging Portal to make it as useful to you as possible.

Think Aloud Method

We are very interested in hearing your thoughts and options while you read the [evidence summary (ES)/blog]. So, as you read through the [ES/blog], I would like you to share your thoughts and opinions by thinking aloud. What I mean by think aloud is to tell me everything you are thinking: say what you're looking at, what catches your eye, what you find confusing. Whatever you are thinking to yourself as you read the [ES /blog], just say it out loud. Just act as if you are speaking to yourself. This lets me know right away about things you are noticing and reacting to while it's still fresh in your mind. Once you have shared a thought aloud, just keep on reading. If you are silent for any long period of time, I will prompt you by saying "please keep talking". There are no right or wrong thoughts; everything you think is okay to say aloud. This will be a big help to us.

Follow-up questions/prompts

Why did you choose this [ES/blog] to review?

What were your first impressions of the [ES/blog]?

Have you been looking for documents like this one?

What did you think about the amount of information provided?

What did you think about how the [ES/blog] is presented (layout)?

Overall, how *clear* (easy to understand) was the information?
Did you learn something?
What was the *key message* of the [ES/blog]?
Were there words that you did not understand?

If you found this on your own, what would you do with the [ES/blog]?
Would you forward or share this [ES/blog] post with friends, family or your doctor?

Will you plan to read any other [ES/blog]s on your own?

How *useful* (relevant) was the information to you or people like you?
What will you do with the information?

Did you notice the related content?

Do you *trust* the information in the [ES/blog]?

Do you have any suggestions to *improve* the [ES/blog]s?

Do you have any other comments?

Evaluation of next [ES/blog]

I would now like to have your feedback on the next [ES/blog] that you selected.
