# Supplementary material for: User Experiences of the McMaster Optimal Aging Portal’s Evidence Summaries and Blog Posts: Usability Study
Source: JMIR Hum Factors. 2016 Aug 19;3(2):e22. doi: 10.2196/humanfactors.6208 (PMC5010647; doi:10.2196/humanfactors.6208)
Supplement: Multimedia Appendix 2 [file humanfactors_v3i2e22_app2.pdf]

User Experiences of the McMaster Optimal Aging Portal's Evidence Summaries and Blog Posts: Usability Study

Appendix 2: Themes and quotes related to elements of the user experience

User experience element	Theme	Exemplar Quotes
**Desirability**		
	Personal relevance	*When I read through the background of this I am trying to decide if it applicable to my grandfather. Because if it isn't, I would stop reading.* (A-13)
		My aunt was in a nursing how a number of years ago and I felt that she was overmedicated. I could not get a clear explanation as to why she was immediately prescribed a mood altering drug, even before seeing a doctor. Since I have no one at home, I expect I will be going into a nursing home one day. I hope they have this sort of thing under control when it is time for me to go into a nursing home. (I-04)
		I personally know this to be exactly true. What is lovely is that you can just pick up and walk. Now, if you have to meet a group, you just can't just pick up and walk. You've got to meet a group at a certain date. But, that is just for me, because I have walked now for at least 15 years, to reduce cholesterol, to keep my blood sugar down. What I was doing was going to the gym in the wintertime so I was able to keep everything where it should be. We had a lovely summer and so I was able to walk a lot, and I noticed my pants are fitting me better even though I have not lost any weight. So, it certainly does something, it tightens you up or something, which is wonderful. (I-08)

		I chose this ES about fall prevention because my grandmother fell and broke her hip, and my mother fell and broke her hip, I am assuming that is probably what will happen to me. (A-17)
		The back pain thing is part of aging I think and maybe a lot of it you can't avoid just because your spine's been taking care of you for so many years, but I think it is a nice thing to know that maybe yoga could help. (I-08)
		This fits with a recent medical visit. At the doctor's office, there was a sign saying if you had more than one question for the doctor, make another visit. I was horrified by that. It does not make sense to have one question per visit when you are dealing with an aging problem. (A-20)
		I am surprised that the interventions had no impact on non-fatal and cardiovascular events. I am sort of surprised by that. (I-01)
		I probably wouldn't read this because I know about that already, but there are a lot of people who don't. (A-20)
**Understandability**		
	Language comprehension	*The individual words are fine. It is just that the phrasing seems like it is intended for professionals, not me.* (A-22)
		I don't understand that. What do you mean by "Playing music in nursing homes may reduce resistance to care?" Do you mean the staff won't help them when they are playing music? (I-05)

		Will an average 60 year old want to read on based on the title? They would have to be very keen. You need something that grabs you. The title is just a statement, but a question would grab me because I would want to know the answer, yes or no. (A-18)
	Grasping the message	*Reinforced my belief that you should get the flu vaccine.* (I-01)
		It's telling me to get exercising. (I-02)
		The one thing that I would want in the conclusion that I should takeaway – What should I do differently? (I-01)
		I would like to actually re-read it and may be think more about it. (I-02)
	Dealing with uncertainty	*It does not state that they are wonder drugs, and that is what I like about it.* (I-09)
		You can't control everything in a study. There is a limitation to science and this is one of the limitations, so I understand that and I am not frustrated by that, but you know, other people might read that and think, what is the point? I know what is a placebo effect is but I often wonder if everybody does. (A-16)
**Usability**		
	Volume of information	*One page is long enough. Any more would be too long*. (A-14)
		So it's nice that it's broken up into these giant titles that kind of breaks it up. And it can let me skip right to what I want to see, so if I wanted I could jump to the conclusion. (A-13)
		If I wanted more information, I would go to related content. (I-01)
		I always look to see how long it is before I commit to reading it. (A-19)
	Use of numbers	*I really like the chart.* (A-21)<br>*I want to know the age range of participants.* (I-03)
		It only gave me statistics, which I don't care about. It did not tell me anything about it, what to look for or how to prevent it. (I-07)
		I don't care much about the statistics or how they got them. I care that I keep up a proper

		speed for my aging to keep in shape. (A-17)
**Usefulness**		
	Intention to use the information	*The blog didn't really tell me anything. They are pretty vague kind of suggestions to me. A little bit more meat, you know some more concrete suggestions might be helpful.* (A-15)
		Now that I have read this, I have to decide what I will do about it. (I-08)
		It shows there is not set amount of sleep. Tells when to seek medical advice. It is useful. (A-16)
		I will come back to the website when I have questions or time. (A-17)
	Facility for sharing	*I would share this evidence summary. Actually, I am going to talk to a friend about getting her husband with dementia out walking more*. (I-02)
		I would share with friends who are not convinced about the value of flu shots. (A-19)
		You should promote these in doctor's offices. (I-06)
